# snRNA‐Seq and Spatial Transcriptome Reveal Cell–Cell Crosstalk Mediated Metabolic Regulation in Porcine Skeletal Muscle

**DOI:** 10.1002/jcsm.13752

**Published:** 2025-03-13

**Authors:** Liu Guo, Mengmeng Han, Junfei Xu, Wenyue Zhou, Hanjing Shi, Sisi Chen, Weijun Pang, Xing Zhang, Yehui Duan, Yulong Yin, Fengna Li

**Affiliations:** ^1^ Laboratory of Animal Nutritional Physiology and Metabolic Process, Key Laboratory of Agro‐Ecological Processes in Subtropical Region, Institute of Subtropical Agriculture Chinese Academy of Sciences Changsha China; ^2^ University of Chinese Academy of Sciences Beijing China; ^3^ Laboratory of Animal Fat Deposition and Muscle Development, College of Animal Science and Technology Northwest A&F University Yangling China; ^4^ The National and Local Joint Engineering Laboratory of Animal Peptide Drug Development, College of Life Sciences Hunan Normal University Changsha China; ^5^ College of Advanced Agricultural Sciences University of Chinese Academy of Sciences Beijing China

**Keywords:** intercellular interaction, metabolic regulation, microenvironment, skeletal muscle, snRNA‐seq, spatial transcriptome

## Abstract

**Background:**

Cell–cell crosstalk between myogenic, adipogenic and immune cells in skeletal muscle to regulate energy metabolism and lipid deposition has received considerable attention. The specific mechanisms of interaction between the different cells in skeletal muscle are still unclear.

**Methods:**

Using integrated analysis of snRNA‐seq and spatial transcriptome, the gene expression profile of *longissimus dorsi* (LD) muscle was compared between adult Taoyuan black (TB, obese, native Chinese breed) and Duroc (lean) pigs.

**Results:**

TB pig had more intramuscular fat (IMF) deposition (3.91%, *p* = 0.0244) and higher slow myofiber proportion (17.13%, *p* < 0.0001) compared with Duroc pig (IMF, 2.38%; slow myofiber, 6.92%) at the age of 180 days. We identified eight cell populations in porcine LD muscle. Five subpopulations of myonuclei and 10 subclusters of fibro/adipogenic progenitors (FAPs) were defined by marker genes. CellChat analysis revealed that communication between immune cells and other cells via the BMP and EGF signalling pathway was only observed in Duroc and not in TB pig. Both snRNA‐seq and spatial transcriptome pointed out that FAPs are the important source of secretory proteins. A total of 35 upregulated and 23 downregulated differentially expressed genes (DEGs) were annotated as secretory, one upregulated and 36 downregulated secretory DEGs were identified between TB and Duroc pigs in FAPs by snRNA‐seq and FAPs‐high regions by spatial transcriptome, respectively. The distribution of FAPs was accompanied by the divergent myofiber‐type composition. The expression level of slow myofiber marker gene (*MYH7*) was higher in both FAPs‐high and FAPs‐low regions of TB compared with Duroc pig (*p* < 0.0001), and expression level of fast myofiber maker gene (*MYH1*) was upregulated in FAPs‐high region of Duroc compared with FAPs‐high region of TB (*p* < 0.0001) and FAPs‐low region of Duroc pig (*p* = 0.0002). The metabolic differences of myofibers between TB and Duroc pigs were mainly concentrated in energy, lipid and nitrogen metabolism‐related pathway (*p* < 0.05). The significant correlation (*R* > 0.4, *p* < 0.05) between secretory and metabolism‐related DEGs with spatial aggregation was verified by regression analysis for random region extraction (area of 25 spots, *n* = 400) from spatial transcriptome, and we speculated that the alteration of secretory proteins forming the microenvironment might regulate myofiber metabolism via target genes such as *IRS1*, *PLPP1* and *SLC38A2*.

**Conclusions:**

Our study provides new insights into skeletal muscle microenvironment that contributes to metabolic regulation and new methods and resources to study cell–cell communication in skeletal muscle.

## 1 Introduction

Skeletal muscle is the largest locomotor organ of the whole body, maintaining physiological activity and system energy homeostasis [1]. Skeletal muscle metabolism and energy expenditure reflected in intramuscular fat (IMF) content and myofiber type composition are strongly correlated with obesity, ageing and metabolic diseases [2], which are considered to be enormous health problems currently facing around the world [3, 4]. Analogously, compared with Duroc (lean) pig, Taoyuan black (TB, native Chinese breed, obese) pig has higher IMF, greater proportion of slow‐twitch myofiber and more abundant adipose tissue [5, 6, 7], which is an ideal model with hereditary stability to study the mechanism of metabolic diseases for humans [8, 9, 10, 11].

Skeletal muscle is also an important secretory organ that releases ‘myokines’, which act as mediators in communication with other tissues to play a role in metabolic regulation via endocrine [12, 13]. Furthermore, all secretory factors released from various cell populations into the extracellular fluid constitute the microenvironment in skeletal muscle, which plays a critical role in the regulation of cell proliferation, differentiation and metabolism via paracrine by complex signalling pathways [14, 15]. Many cytokines with metabolic regulation through interactions between cells and tissues have been discovered [16, 17, 18]. For example, muscline was one of the novel myokines screened by RNA‐seq between fat‐ and lean‐derived skeletal muscle from humans and mice, promoting thermogenesis via Tfr1/PKA signalling in beige fat [19]. Proteomic of extracellular fluid isolated from skeletal muscle identified a novel myokine, prosaposin, that was more abundant under exercise and cold exposure to accelerate oxidative metabolism in adipocytes [20]. However, majority of studies explored novel secretory proteins based on bulk RNA‐seq or proteome [21], whose results only displayed the average expression level of the tissue, which is made up of a variety of cells. Therefore, the mechanism of intercellular interaction mediated by cytokines with clear source and target in skeletal muscle is still limited. scRNA‐seq and spatial transcriptomics techniques achieve the acquisition of gene expression profiles at the single‐cell and spatial levels, which is an ideal method to investigate the mechanism of cell–cell interaction mediated by niche [22, 23, 24]. Single‐cell and spatial transcriptome has been progressively used to identify key secretory proteins and uncover the mechanism of skeletal muscle metabolic diseases in mice model [25, 26, 27]. However, using integrated analysis of single‐cell and spatial transcriptome to reveal the different metabolic regulation mediated by intercellular interaction in adult porcine muscle has not yet been reported.

Therefore, we conducted integrated snRNA‐seq and spatial transcriptome analysis of the *longissimus dorsi* (LD) muscle between adult TB and Duroc pigs, which revealed a discrepancy in the expression profile between TB and Duroc pigs at the age of 180 days with cellular heterogeneity and spatial specificity. The results revealed major differences in signalling communication involving immune cells centred on the BMP and EGF pathways between TB and Duroc pigs. And fibro/adipogenic progenitors (FAPs) were critical source of secretory proteins in skeletal muscle. Furthermore, many novel cytokines with distinct origins that contribute to the skeletal muscle microenvironment involved in intercellular interaction were identified, whose correlation with myofiber type marker genes and metabolism‐related differentially expressed genes (DEGs) was verified by regression analysis. Our study highlights important contribution to metabolic regulation of interaction between various cell populations in skeletal muscle and provides potential secretory genes to be diagnostic and intervention targets for metabolic diseases in humans and meat quality in pigs.

## 2 Methods

### 2.1 Animal Model and Experimental Design

The care and handling of the pigs used in this study followed the standards of the Animal Care and Use Committee of the Institute of Subtropical Agriculture, Chinese Academy of Sciences (No. ISA‐2022‐0001). Ten TB and 10 Duroc pigs were fed same diet in separate rooms during the age of 30–180 days. All pigs were male and fed in comfortable environment under constant temperature (22°C) and humidity (60%) with three times feed a day and water for free intake.

### 2.2 IMF Detection

IMF content of skeletal muscle was detected using the Soxhlet extraction method referred to in previous studies [28].

### 2.3 Immunofluorescence (IF) Staining and Analysis

For IF staining of LD muscle, using primary antibody against slow skeletal myosin heavy chain (Abcam, ab234431, 1:400) and CY3‐conjugated second antibody (Boster, BA1032, 1:200); primary antibody against fast skeletal myosin heavy chain (Abcam, ab51263, 1:400) and 488‐conjugated second antibody (Boster, BA1126, 1:200). DAPI was used to label nuclei with fluorescence. The fluorescence images were visualized using confocal laser scanning microscopy (3DHISTECH, Pannoramic MIDI). The Image J software was used to quantify the number of myofibers identified as slow and fast type from five fields, IF staining of skeletal muscle, myofiber type identification and statistical analysis were referred to previous study [29].

### 2.4 Single‐Nucleus Isolation and snRNA‐Seq Analysis

About 0.1‐g LD muscle tissue was collected from three TB and three Duroc pigs for snRNA‐seq analysis; nuclei isolation was performed by LC‐Bio Technology Co. Ltd. (Hangzhou, China) according to the standard protocol described in previous research [30]. Single‐nuclei suspensions were loaded to 10× Chromium to capture 8000 single cells according to the manufacturer's instructions of 10× Genomics Chromium Single‐Cell 3′ kit (V3). The cDNA amplification and library construction steps were performed by LC‐Bio Technology Co. Ltd. (Hangzhou, China) according to the standard protocol. Libraries were sequenced on an Illumina NovaSeq 6000 sequencing system (150 bp) at a minimum depth of 20 000 reads per cell.

Raw data were demultiplexed and converted to FASTQ format using Illumina bcl2fastq software (v 5.01). A total of single cells captured from six samples were performed using 10× Genomics Chromium Single Cell 3′Solution. Cell Ranger was used to process the raw data and align to the reference genome (ftp://ftp.ensembl.org/pub/release‐108/fasta/sus_scrofa/dna) and quantify the expression of transcripts in each cell. The Cell Ranger output was loaded into Seurat (v 4.1.1) for dimensional reduction, clustering and analysis of snRNA‐seq data. Overall, 64 851 nuclei were filtered by doublefinder and quality control threshold: All genes expressed in less than one cell (default parameters: 1 cell) were removed, the number of genes expressed per cell was over 500, and the percent of mitochondrial DNA‐derived gene expression was < 25% (Table S1). A total of 58 671 nuclei were used for downstream analysis.

### 2.5 Spatial Transcriptome Analysis

Approximately 5 × 5 × 5 mm^3^ of LD muscle from one TB and one Duroc pigs were flash frozen and embedded in OCT (SAKURA, 4583) using isopentane (China National Medicines Corporation Ltd., GMA004582)–liquid nitrogen method. The embedding box was wrapped in aluminium foil and stored at −80°C for spatial transcriptome. Spatial transcriptome was performed by LC‐Bio Technology Co. Ltd. (Hangzhou, China) according to the standard protocol. OCT blocks with LD muscle were sectioned at 10‐μm thickness and attached to the Visium slides in the size of 6.5 × 6.5 mm; H&E images were taken by a fluorescence and tile scanning microscope (Olympus Fluoview 1000). Then, the slides were permeabilized for 24 min (Figure S1), and after tissue removal, the library was generated according to the protocol presented by 10× Genomics.

Sequenced spatial transcriptomics library and UMI counts in each spot were processed and aligned to reference genome (ftp://ftp.ensembl.org/pub/release‐108/fasta/sus_scrofa/dna) using Space Ranger software (v 2.0.0) from 10× Genomics. Spots not covered by tissue and detected by Space Ranger and further filtered the UMI count matrices were eliminated. Then, the filtered UMI count matrix was analysed using the R package ‘Seurat (v 4.1.0)’, and regularized negative binomial regression (SCTransform) was used to normalize UMI count matrices. Two matrices from TB and Duroc pig slices were merged to analyse them together.

### 2.6 Bioinformatics Analysis

To identify the cluster of all nuclei, we next reduced the dimensionality using Seurat and t‐SNE to project the nucleus into 2D space. Marker genes for each cluster were identified with the Wilcoxon rank sum test (default parameters are ‘bimod’: Likelihood‐ratio test) with default parameters via the FindAllMarkers function in Seurat. This selects markers genes that are expressed in more than 10% of cells in a cluster and average|log_2_(foldchange)| > 0.26 (default parameters: 0.26). CellChat and CellphoneDB were used to identify ligand–receptor interaction and cell–cell communication networks from snRNA‐seq data [31, 32]. Differential expression analysis was performed using the R package ‘DESeq2 (v 1.46.0)’ under condition *p* < 0.05 and|log_2_(foldchange)| > 0.26. All DEGs were blasted to subcellar locations from the UniProt database (https://www.uniprot.org/) to estimate whether it encodes secretory proteins. Gene Ontology (GO) (http://www.geneontology.org/) and Kyoto Encyclopaedia of Genes and Genomes (KEGG) (https://www.kegg.jp/) enrichment analysis was used to explore the function of DEGs, which were performed by ‘phyper’ based on Hypergeometric test. The significant levels of terms and pathways were corrected by *Q* value with a rigorous threshold (*Q* < 0.05). In order to verify the correlation between the DEGs annotated as secretory initially screened and the DEGs related to the metabolism of myonuclei, we randomly sampled 400 regions with equal area (25 spots) from spatial transcriptome in TB and Duroc samples and then conducted regression analysis of gene expression profiles by ‘Pearson’ correlation analysis with R package ‘psych (v 2.4.12)’.

### 2.7 Statistical Analysis

Experimental data are presented as the mean ± standard deviation (SD), and comparisons between TB and Duroc pigs were performed by Student's *t*‐test. *, **, *** and **** represented significant difference at *p* < 0.05, *p* < 0.01, *p* < 0.001 and *p* < 0.0001. GraphPad Prism (v 9.5.0) and R software (v 4.1.3) were used for data analyses and visualization.

## 3 Results

### 3.1 snRNA‐Seq Identifies Cell Populations in Porcine LD Muscle

**FIGURE 1:**
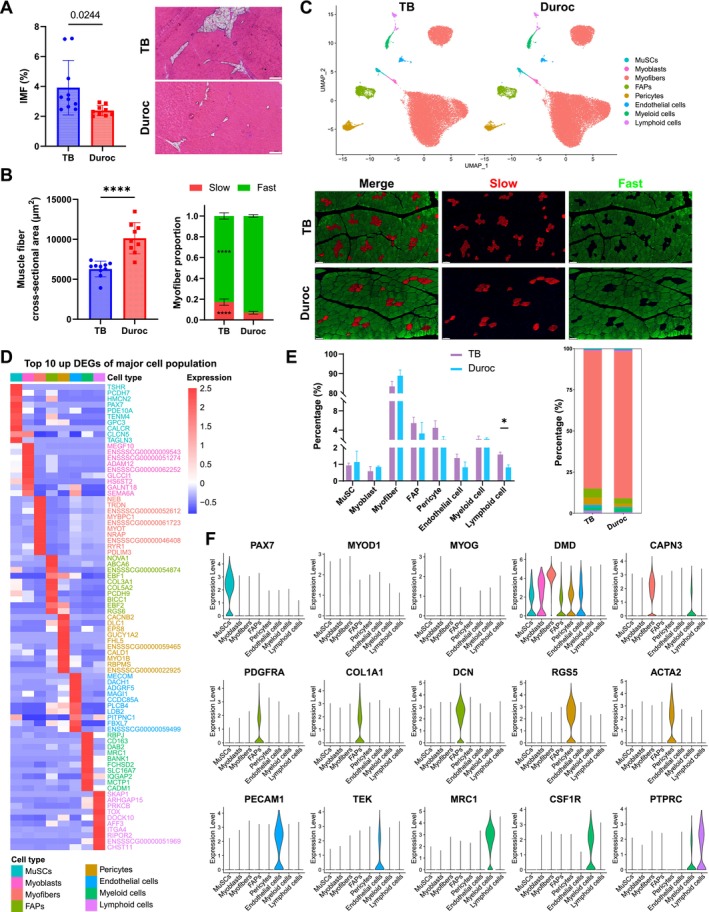
snRNA‐seq identifies cell populations in LD muscle of TB and Duroc pigs. (A) IMF content and frozen sections stained by H&E of LD muscle at 180 days, significantly different between the two breeds (*p* < 0.05) analysed by *t*‐test, scale bars: 200 μm (*n* = 10). (B) Myofiber cross‐sectional area and proportion of myofiber type in LD muscle of TB and Duroc pigs at 180 days, ****significantly different between TB and Duroc pigs (*p* < 0.0001) analysed by *t*‐test. IF staining of slow‐ and fast‐twitch myofibers, scale bars: 100 μm, (*n* = 3). (C) Dimensionality reduction via UMAP of nucleus extracted from LD muscle of TB and Duroc pigs (*n* = 3). (D) Differential gene expression analysis showing the top 10 DEGs for each cell population. (E) Proportion of cell populations in LD muscle of TB and Duroc pigs, *significantly different between TB and Duroc pigs (*p* < 0.05) analysed by *t*‐test. (F) Violin plot of marker gene expression in each cell population.

TB pig, as a typical obese pig model, had higher IMF (*p* = 0.0244) and smaller myofiber cross‐sectional area (*p* < 0.0001) in LD muscle than Duroc pig, which was regarded as classical lean pig. The proportion of slow‐twitch myofibers was higher (*p* < 0.0001), whereas fast‐twitch myofibers had a lower percentage (*p* < 0.0001) in TB than in Duroc pig (Figure 1A,B). Eight cell populations including muscle stem cells (MuSCs) (*PAX7*), myoblasts (*MYOD1*, *MYOG*), myofibers (*DMD*, *CAPN3*), FAPs (*PDGFRA*, *COL1A1*, *DCN*), pericytes (*RGS5*, *ACTA2*), endothelial cells (*PECAM1*, *TEK*), myeloid cells (*MRC1*, *CSF1R*) and lymphoid cells (*PTPRC*) were identified based on the expression of specific marker genes (Table S2), the top 10 significant marker genes of each population were displayed, and proportion of lymphoid cells was higher in TB than in Duroc pig (*p* < 0.05) (Figure 1C–F).

According to known gene markers, we further identified five subpopulations of myonuclei including type 1 (*MYH7*), type 2a/x (*MYH2*), type 2b (*MYH4*), type 2x (*MYH1*) and NMJ (*ABLIM2*) (Figure S2A–C and Table S2). Phylogenetic trees of the myogenic cell populations indicated MuSCs generated to myoblasts and then developed to type 2b myofibers, which branched with two fates including maintaining fast myofibers (type 2x) and transforming into slow myofibers (type 1) through type 2a/x (Figure S2D,E). Compared with fast‐twitch myonuclei (type 2a/x, 2b and 2x), slow‐twitch myonuclei (type 1) presented more active fatty acid metabolism, oxidative phosphorylation and less glycolysis (Figure S2F).

We identified 10 subpopulations of adipogenic cells based on known marker genes (Table S2). Committed preadipocytes that had high expression of *LPL* and *VCAM1*, also as a marker gene of preadipocytes, interstitial cells with the highest expression level of *THY1*, tenocytes (*THY1*, *CD34*), MYOC^+^ FAPs (*MYOC*), MT‐rich FAPs (*CYTB*, *COX2*), fibroblasts presented characteristics of fibrogenesis (*COL1A1*, *COL11A1*), transitional FAPs (*APOD*), adipocytes with high expression of genes related to lipid storage (*FABP4*, *ADIPOQ*, *PPARG*) and myofibroblasts (*ACTA2*) were defined according to their specific gene expression. Extraordinary, muscle‐specific genes (*ACTA1*, *MYOT*, *MYBPC1*) were highly expressed in myocyte‐like FAPs, which occupied a higher proportion in Duroc than in TB pig (*p* < 0.1) with unknown function (Figure S3A–C). FAPs of TB pig had higher expression level of the genes for adipogenesis (*CEBPB*, *PPARG*, *PPARGC1A*, *FASN*, *ACSL1* and *LPIN1*) and lipolysis (*PNPLA2*, *LPL*, *MGLL*, *LDAH* and *DDHD2*) compared with Duroc pig (Figure S3D–F).

### 3.2 CellChat Analysis Reveals Interaction Between Myogenic, Adipogenic and Immune Cells

**FIGURE 2:**
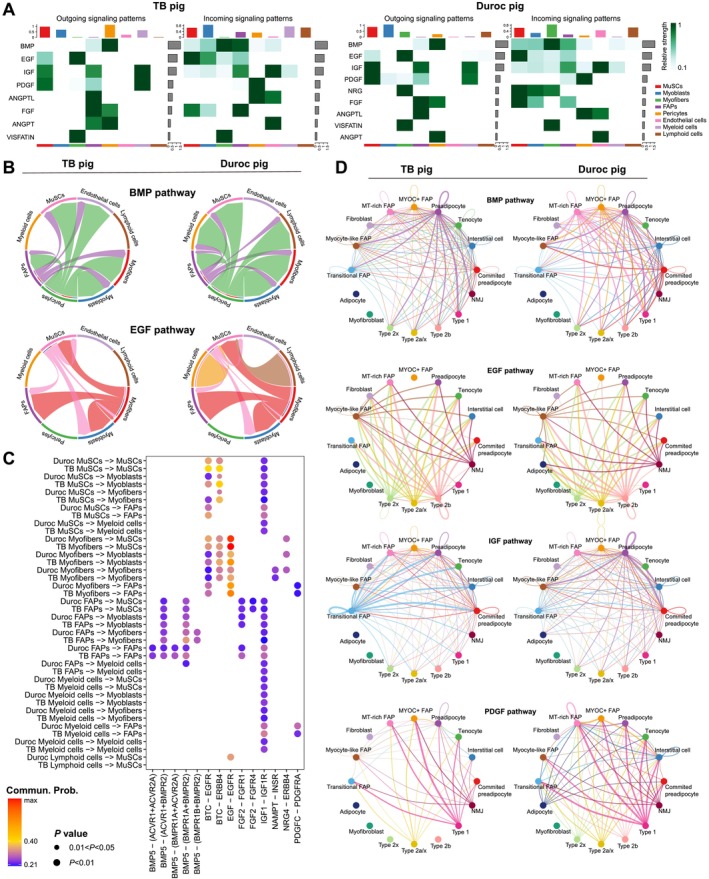
CellChat analysis reveals difference of signal communication in LD muscle between TB and Duroc pigs. (A) Heatmap showing outgoing and incoming communication patterns between cell populations in TB and Duroc pigs. (B) Interactions on BMP and EGF pathway between major cell populations in TB and Duroc pigs. (C) An interaction is indicated as colour‐filled circle at the cross of interacting cell population (y‐axis) and a ligand‐receptor pair (x‐axis), colour represents the means of the average expression level of the interacting pair analysed by CellChat. (D) Interactions on BMP, EGF, IGF and PDGF pathway between subpopulations of myofibers and FAPs in the TB and Duroc pigs.

We performed a ligand–receptor interaction analysis using CellChat to display the difference in signalling pathway networks between TB and Duroc pigs. The number of interactions between all cell populations suggested that FAPs were the hub of communication in skeletal muscle, which was proved by both CellChat and CellPhoneDB analysis. Communication between immune (myeloid and lymphoid) cells and other cell populations by BMP and EGF pathway only appeared in Duroc pig (Figures 2A,B and S4A,B). The interaction from myofibers to other myogenic cells via NRG4 and ERBB4, FAPs to myeloid cells via BMP5 and BMPR1A/2 and lymphoid cells to MuSCs via EGF and EGFR was only observed in Duroc pig; MuSCs provided BTC to EGFR in myofibers that existed in TB rather than in Duroc pig (Figures 2C and S4C). We also conducted cell interaction analysis between subpopulations of FAPs and myofibers. The BMP secreted form FAPs (committed preadipocytes, interstitial cells, MYOC^+^ FAPs and MT‐rich FAPs) was stronger in Duroc compared with TB pig, and this trend almost acted on all myofiber subpopulations. The EGF from fast myofibers (type 2a/x, 2b and 2x) in Duroc pig targeted to adipocytes, but this phenomenon was not observed in TB pig, and myofibers (type 2b and 2x)–derived EGF was only received by MYOC^+^ FAPs in Duroc pig. A stronger IGF signal pathway from preadipocytes targeted to myofibers was observed in Duroc pig. Besides, interaction between subpopulations of FAPs was also different between the two breeds, IGF from transitional FAPs to other clusters was more apparent in TB than in Duroc pig. The PDGF secreted from adipocytes, as well as type 2b and 2x myofibers, were only presented in Duroc rather than in TB pig (Figure 2D).

### 3.3 Spatial Transcriptome on LD Muscle Defines Cell Clusters Based on snRNA‐Seq

**FIGURE 3:**
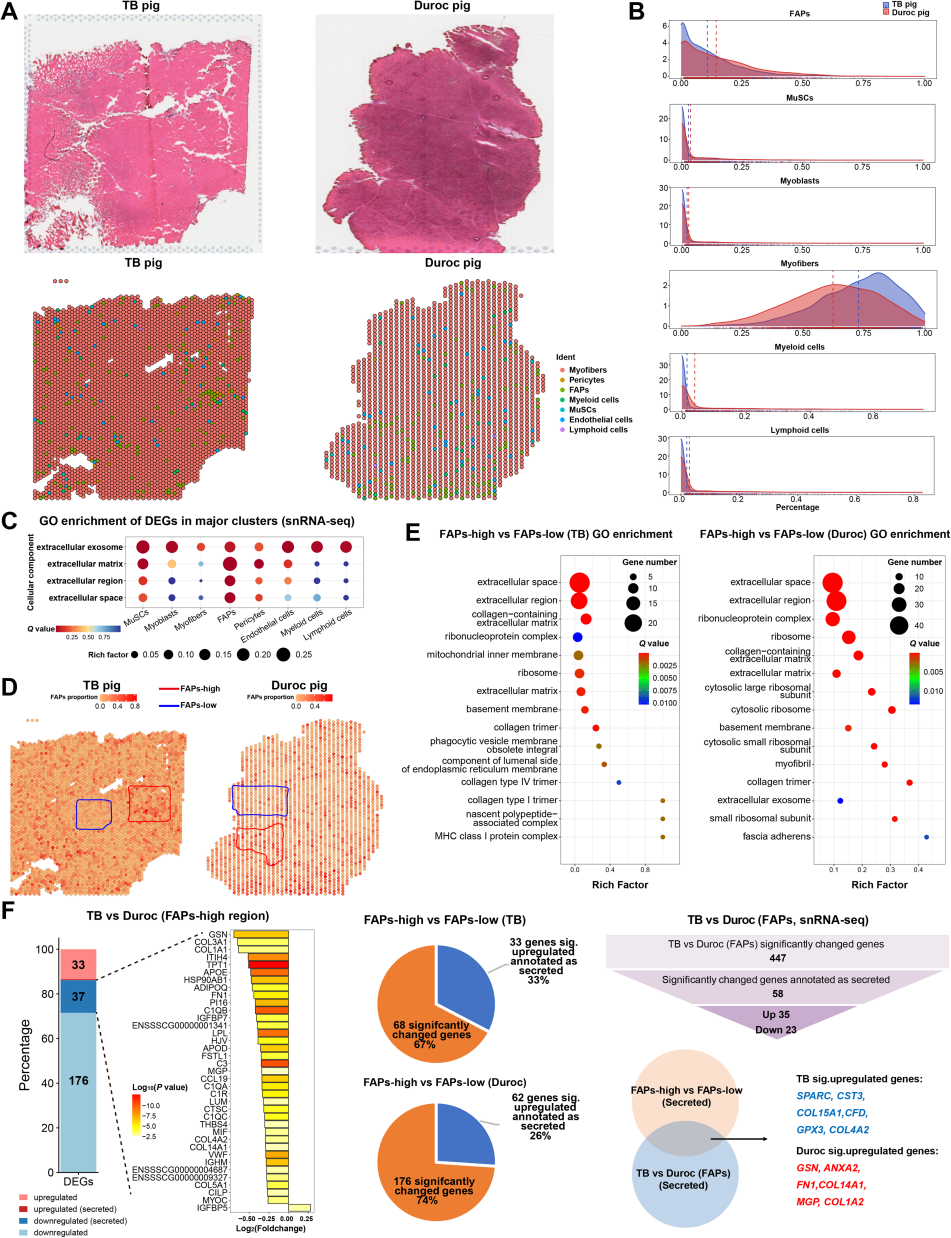
FAPs are important source of secretory proteins. (A) H&E staining and cell populations in spatial transcriptome identified by transposed convolution (Intergrated ANOT) mapping on snRNA‐seq (*n* = 1). (B) Distribution of myogenic, adipogenic and immune cells in LD muscle of TB and Duroc pigs from results of transposed convolution (SPOTlight). (C) GO enrichment analysis showing the cellular component enrichment of DEGs for each cell population. (D) Spatially resolved proportion of FAPs in each spot, the colour represents percentage of FAPs in each spot. Blue represents FAPs‐low region, and red represents FAPs‐high region. (E) GO enrichment analysis showing the cellular component of up‐DEGs in FAPs‐high compared to FAPs‐low regions. (F) Differential gene analysis and secretory annotation between TB versus Duroc pig (FAPs‐high region), FAPs‐high versus FAPs‐low regions and TB versus Duroc pig (FAPs from snRNA‐seq).

We used capture probe‐based spatial transcriptome (Visium, 10× Genomics) to gain a spatial expression profile of LD muscle, which provided spatially resolved gene expression analysis limited in resolution by 55‐μm‐diameter spot size (Figure 3A); a total of 3982 and 1813 spots with 921 and 1430 median genes per spot were obtained in TB and Duroc pigs and divided into clusters on the basis of gene expression profile (Figure S5). Then, we defined cell populations in spatial transcriptome mapping on snRNA‐seq data by two methods of transposed convolution: Intergrated ANOT and SPOTlight. The percentage of cell populations in all spots was calculated according to the results of SPOTlight, and the distribution of myogenic, adipogenic and immune cells was displayed in Figure 3B.

### 3.4 FAPs as a Critical Source of Secretory Proteins That Mediate Intercellular Communication

Based on the results of snRNA‐seq, we found that FAPs‐specific upregulated genes were significantly annotated to the extracellular by GO enrichment analysis (Figure 3C). We delineated region with high and low proportions of FAPs in TB and Duroc pigs, which were defined as FAPs‐high (red) and FAPs‐low (blue) regions (Figure 3D). Both in TB and Duroc pigs, the upregulated DEGs in the FAPs‐high versus FAPs‐low region were enriched into extracellular‐related cellular component, indicating the FAPs were important source of secretory proteins involved in intercellular communication (Figure 3E). Therefore, we identified FAPs‐derived secretory proteins via three comparisons and blast with subcellar location information from the UniProt database: TB versus Duroc (FAPs‐high region), FAPs‐high versus FAPs‐low region, TB versus Duroc (FAPs from snRNA‐seq). A total of 37 DEGs annotated as secreted were identified in TB versus Duroc (FAPs‐high region), all of which except *IGFBP5* were upregulated in Duroc than in TB pig. Twelve common secretory genes screened from FAPs‐high versus FAPs‐low region and TB versus Duroc (FAPs from snRNA‐seq), *SPARC*, *CST3*, *COL15A1*, *CFD*, *GPX3* and *COL4A2* were upregulated in FAPs of TB pig, *GSN*, *ANXA2*, *FN1*, *COL14A1*, *MGP* and *COL1A2* with higher expression in Duroc pig attracted our attention (Figure 3F and Tables S3–S6).

### 3.5 Changes in Secretory Gene Expression Profiles of FAPs Between TB and Duroc Pigs

**FIGURE 4:**
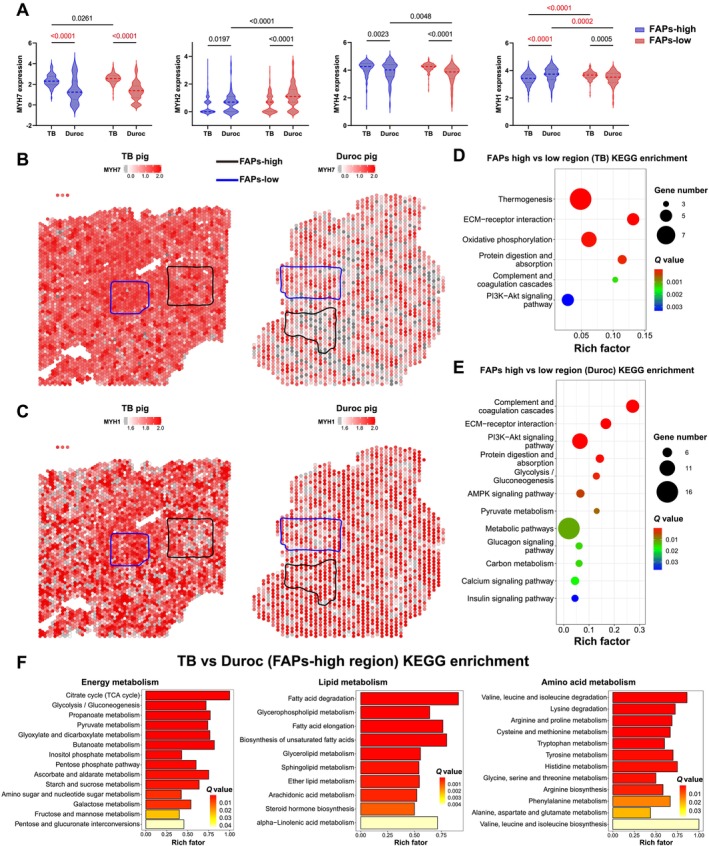
Myofiber type composition and metabolic heterogeneity related to FAPs distribution. (A) Violin plots of myofiber type maker genes in spots from FAPs‐high and FAPs‐low regions of TB and Duroc pigs, significantly different between groups (*p* < 0.05) analysed by two‐way ANOVA. (B) and (C) Spatially resolved gene expression of *MYH1* and *MYH7* in LD muscle of TB and Duroc pigs. Blue represents FAPs‐low region, and black represents FAPs‐high region, the colour of spots represents the expression level of genes. (D) and (E) KEGG enrichment analysis showing metabolism‐related pathway enriched in FAPs‐high versus FAPs low regions of TB and Duroc pigs, *Q* value < 0.05 means significant, rich factor is the percentage of DEGs enriched in pathway to total genes involved in pathway. (F) KEGG enrichment analysis of DEGs showing metabolic difference between TB versus Duroc (FAPs‐high region), *Q* value < 0.05 means significant, rich factor is the percentage of DEGs enriched in pathway to total genes involved in pathway.

Differential analysis of FAPs from snRNA‐seq between the two breeds demonstrated huge difference in MAPK, cGMP‐PKG and protein digestion and absorption signalling pathways (Figure S6A,B). Secretory DEGs of FAPs and subpopulations are shown in Figure S6C,D. To verify whether the FAP distribution is associated with the myofiber type, we showed spatial expression profile of *MYH7* and *MYH1*, which was regarded as the marker genes of slow and fast myofibers. *MYH1* was downregulated in FAPs‐high versus FAPs‐low region of TB pig (*p* < 0.0001), and the average expression level of *MYH1* was higher (*p* = 0.0002) in FAPs‐high compared with FAPs‐low region of Duroc pig. Meanwhile, *MYH7* was upregulated (*p* < 0.0001), and *MYH1* presented lower expression (*p* < 0.0001) in FAPs‐high region of TB compared with Duroc pig, demonstrating strong correlation between FAPs and myofiber type (Figure 4A–C).

The DEGs between FAPs‐high versus FAPs‐low region of TB pig were enriched in thermogenesis, oxidative phosphorylation and protein digestion and absorption, and the difference of metabolism concentrated on P13K‐Akt signalling pathway, cardiac muscle contraction, protein digestion and absorption, and glycolysis/gluconeogenesis between FAPs‐high versus FAPs‐low region of Duroc pig (Figure 4D,E), indicating metabolic heterogeneity associated with FAPs distribution, which is caused by different skeletal muscle microenvironments, whose DEGs annotated as secreted was shown in Figure S6E. Furthermore, we also noticed significant metabolic differences in energy (TCA cycle, glycolysis/gluconeogenesis), lipid (fatty acid degradation, glycerophospholipid metabolism) and amino acid (valine, leucine and isoleucine degradation, lysine degradation and arginine and proline metabolism) metabolism in FAPs‐high regions between the two breeds (Figure 4F).

### 3.6 Difference in Metabolic and Secretory Gene Expression Profiles of Myonuclei Across Species

**FIGURE 5:**
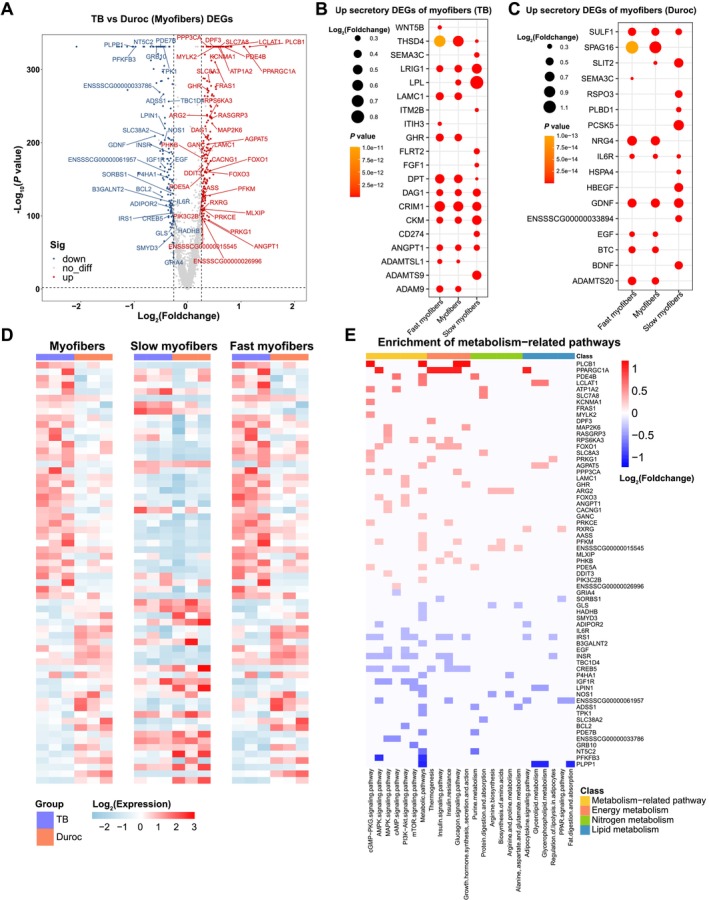
Differences in metabolism and secretory profiles of myonuclei between TB and Duroc pigs. (A) Volcano plot of differential gene analysis of myonuclei between TB and Duroc pigs. (snRNA‐seq), red represents upregulated DEGs, blue represents downregulated DEGs, metabolism‐related DEGs are labelled. (B) and (C) upregulated/downregulated secretory DEGs identified in slow and fast myonuclei between TB and Duroc pigs. (D) The heatmap showing the expression level of metabolism‐related DEGs in slow and fast myonuclei. (E) Pathway analysis of DEGs of myonuclei enriched in metabolism‐related pathway between TB and Duroc pig based on snRNA‐seq.

Since myofibers account for the largest proportion of skeletal muscle, to evaluate differences in the level of metabolism in myonuclei between TB and Duroc pigs, we performed differential analysis and KEGG enrichment analysis of myonuclei between the two breeds (Figure 5A; Table S7). All significant pathways related to metabolism are displayed and divided into three main categories: energy, nitrogen and lipid metabolism (Figure 5E). We also displayed the expression of these DEGs with metabolic regulation in slow and fast myonuclei, the great mass of upregulated DEGs in TB pig had low expression level in slow myonuclei, and the difference almost due to the higher expression in fast myonuclei of TB compared to Duroc pig. But majority of downregulated DEGs in TB pig had a downward trend in both slow and fast myonuclei (Figure 5D).

Not only FAPs but myofibers release secretory proteins to affect systemic metabolism. We identified secretory DEGs of myonuclei between the two breeds to screen novel myokines associated with obesity. The expression of myokines was significantly different in slow and fast myonuclei. *LPL*, *ITM2B*, *FLRT2*, *FGF1*, *CD274*, *ADAMTS9*, *SLIT2*, *PSPO3*, *PLBP1*, *PCSK5*, *HSPA4*, *HBEGF* and *BDNF* had changed expression in slow but not fast myonuclei, whereas *WNT5B*, *LAMC1*, *ITIH3*, *GHR*, *ADAMTSL1*, *ADAM9*, *EGF*, *BTC* and *ADAMTS20* only differed in fast myonuclei between the two breeds. Besides, some myokines had differential expression in both fast and slow myonuclei (Figure 5B,C).

### 3.7 Metabolic Regulation of Cytokines With Explicit Source and Verified by Regression Analysis

**FIGURE 6:**
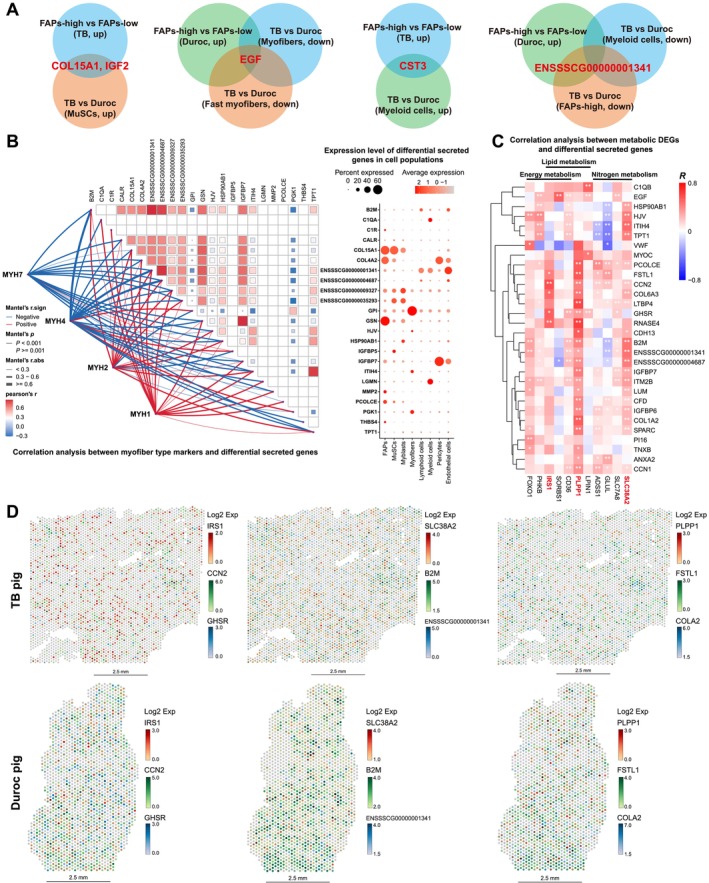
Regression analysis of potential secretory DEGs and metabolism‐related DEGs of myonuclei. (A) Venn diagrams of secretory DEGs from MuSCs, myofibers and myeloid cells identified in snRNA‐seq and spatial transcriptome. (B) Correlation analysis of potential secretory DEGs and myofiber type markers with average expression in major cell populations, potential secretory DEGs were screened from differential analysis in Figure 3F. (C) Correlation analysis of potential secretory DEGs and metabolism‐related genes. Potential secretory DEGs were screened from differential analysis in Figure 3F, and metabolism‐related genes was screened from myonuclei in Figure 5A, *, **significant correlation between genes (*p* < 0.05, *p* < 0.01), the scale bar represents the correlation coefficient (*R*). (D) The spatial aggregation of the top two secretory genes with *IRS1*, *SLC38A2* and *PLPP1* in TB and Duroc pigs, the scale bar represents the expression level of genes.

snRNA‐seq and spatial transcriptome identified several secretory genes with definite origin and significant differences between TB and Duroc pigs. *COL15A1* and *IGF2* were upregulated in FAPs‐high versus FAPs‐low region in TB pig and had higher expression in MuSCs of TB than that of Duroc pig, indicating that MuSCs‐derived *COL15A1* and *IGF2* were connected with the distribution of FAPs in TB pig (Figure S7A and Table S8). *EGF* was identified with higher expression in fast rather than slow myonuclei of Duroc than that of TB pig and upregulated in FAPs‐high versus FAPs‐low region in Duroc pig, which might explain more abundant signal communication mediated by myofibers‐derived EGF targeting to FAPs and immune cells in Duroc pig. As for myeloid cells, *CST3* was upregulated, whereas *ENSSSCG00000001341* was downregulated in TB compared with Duroc pig (Figures 6A and S7B and Table S9).

In order to explore the correlation and spatial aggregation of DEGs related to metabolism in myonuclei (from Figure 5A) and secretory DEGs related to FAPs distribution (from Figure 3F), we extracted the expression profile of 200 regions with an area of about 25 spots in each spatial transcriptome sample via random program. The expression of 23 secretory genes was significantly correlated with markers of myofiber type (*R* > 0.5, *p* < 0.001). As expected, almost all secretory genes showed opposite correlations with marker genes of fast (*MYH1*) and slow (*MYH7*) myofibers. Meanwhile, the explicit origin of these secretory genes was also identified according to the expression level of cell populations. FAPs‐derived *CR1*, *COL15A1*, *COL4A2*, *GSN*, *MMP2* and *THBS4*; MuSCs‐derived *HMGB1*, *IGF2* and *HSP90AB1*; myofibers‐derived *GPI*, *HJV*, *ITIH4* and *PGK1*; and immune cells‐derived *B2M*, *C1QA* and *LGMN* showed significantly negative correlation with *MYH7* and were positively correlated with *MYH1*. Interestingly, only MuSCs‐derived *IGFBP5* presented a significantly positive correlation with the maker gene (*MYH7*) of slow myofiber (Figure 6B), which was consistent with the only *IGFBP5* increased in FAPs‐high region of TB than that of Duroc pig, indicating that MuSCs‐derived *IGFBP5* might promote the proliferation of FAPs. Then, we performed regression analysis between secretory DEGs and metabolism‐related genes, and the expression of these secretory genes in major cell populations is displayed in Figure S7C. *CCN2*, *GHSR*, *RNASE4*, *COL6A3* and *FSTL1* were positively correlated with the expression of *IRS1*, which is a key factor for glucose metabolism and insulin sensitivity. And *VWF* had a negative correlation with the expression of *FOXO1* (*R* > 0.4, *p* < 0.05), which regulated myogenic growth and differentiation mediated by *PAX3*. In addition, *ENSSSCG00000001341*, *B2M*, *HJV*, *ENSSSCG00000004687*, *TPT1* and *ITIH4* showed a significantly positive correlation with genes related to nitrogen metabolism, such as *SLC38A2* and *GLUL* (*R* > 0.4, *p* < 0.05), and *EGF* was positively correlated with *SORBS1* involved in PPAR signalling pathway (*R* > 0.5, *p* < 0.05) (Figure 6C). The spatial aggregation of these genes with significant correlation was observed (Figure 6D).

## 4 Discussion

In this study, we first performed an integrated snRNA‐seq and spatial transcriptome analysis of LD muscle from adult obese and lean pigs, which are ideal research models for ageing, obesity and metabolic diseases.

An important finding is that complex signalling communication between myogenic, adipogenic and immune cells may contribute to the differences in IMF accumulation and myofiber‐type composition in skeletal muscle. The immune cells (myeloid and lymphoid cells) communicated with myogenic and adipogenic cells via BMP and EGF signalling pathway is observed in Duroc rather than in TB pig. Different interaction patterns are also observed in subpopulations of myofibers and FAPs may explain the stronger capacity of lipid deposition in TB pig. Upregulated DEGs of FAPs‐high versus FAPs‐low regions are annotated to extracellular related cellular component both in TB and Duroc pigs, and specific genes expression in FAPs compared with other cell populations are significantly enriched in extracellular. Our research highlights that FAPs are important cell population to release secretory proteins which contribute to the microenvironment of skeletal muscle. Moreover, many differentially expressed myokines and adipokines with explicit source between the two breeds are identified. A total of 12 secretory genes with significant difference between TB and Duroc pigs are identified from both snRNA‐seq and spatial transcriptome. *SPARC* with a higher expression level in TB pig is demonstrated function to regulate IMF deposition in skeletal muscle and correlation with ageing [16]. *CFD* is also identified as a critical gene significantly related to high IMF accumulation from scRNA‐seq of cattle and humans [33]; in our results, upregulated expression level of *CFD* is presented in FAPs‐high versus FAPs‐low region and FAPs of TB compared with Duroc pig. On the contrary, *GSN*, *ANXA2*, *FN1* and *MGP* are upregulated in Duroc than in TB pig. *ANXA2* promotes myoblasts proliferation and skeletal muscle growth by activating PI3K‐mTOR pathway [34], which may explain higher muscle mass and faster muscle growth of Duroc pig. Exercise‐activated *FN1* secreted from muscle is proved to interact with the liver and induces insulin sensitization via α5β1 integrin and IKKa/b‐JNK1‐BECN1 pathway [35]. *MGP* is reported as a novel target for muscle weakness with high expression level in the musculoskeletal system [36].

Also interesting is that the secretory DEGs related to FAPs distribution are closely associated with the marker genes of myofiber‐type and metabolism‐related DEGs of myonuclei. These secretory proteins that constitute microenvironment and participate in cell–cell interactions through paracrine may have spatial aggregation, whose regulatory ability decreases with the increase of spatial distance. Therefore, we did not perform validation for these potential secretory DEGs using traditional methods like qPCR or WB in the whole muscle tissue, which may weaken the regionalized interaction. Innovatively, we conducted a regression analysis of secretory genes and metabolism‐related DEGs by selecting regions from TB and Duroc pigs randomly. A total of 23 secretory genes with explicit origin are significantly associated with marker genes of myofiber‐type *MYH7* and *MYH1* showing the opposite correlation. The differential gene expression profile between the two breeds is mainly concentrated on the pathways related to energy, nitrogen and lipid metabolism, which is consistent with research between young, healthy men and older, metabolically compromised men [37]. The expression of *IRS1*, *SLC38A2* and *GLUL* show the most significant correlation with secretory genes and are related with FAPs distribution. Therefore, we speculate that the different microenvironment of skeletal muscle between TB and Duroc pigs may regulate energy metabolism by targeting *IRS1*, upregulate *SLC38A2* and downregulate *GLUL* to modulate nitrogen metabolism of myofibers and induce *PLPP1* to promote lipolysis.

Our study has several limitations. Firstly, all pigs in this experiment were male, so the influence of sex cannot be ruled out. Secondly, we only observed significant correlation and spatial aggregation between myofiber type marker genes, metabolism‐related genes and many potentially secretory proteins, whose specific metabolic regulatory functions and mechanisms involved in adipose‐muscle tissue crosstalk are still unclear. Therefore, the role of sex in modulating the distribution of myofiber types, metabolic pathways and the intricate crosstalk between adipose and muscle tissue remains an unexplored dimension that future studies should address. Finally, genetic backgrounds of TB and Duroc pigs are very diverse, and it is not clear whether the difference in muscle gene expression revealed by snRNA‐seq and spatial transcriptome between the two breeds is caused by different genetic backgrounds or related to differences in muscle lipid deposition and metabolism. Further verification of expression differences in individuals with extreme muscle lipid deposition within same pig breed or other species (human or mouse) is the future research direction.

In conclusion, our study provides new insights into the regulation of skeletal muscle metabolic reprogramming by different microenvironment consists of secretory proteins, and we emphasize complex signal communication between myogenic, adipogenic and immune cells leads to the difference in IMF deposition and metabolism of skeletal muscle between obese and lean pig models. Above all, many novel secretory proteins involved in cell–cell crosstalk are identified and expected to be regarded as potential diagnostic biomarkers and therapeutic targets for ageing, obesity and metabolic diseases of humans. Finally, our study also develops novel methods and resources for studying the interaction between different cell populations in skeletal muscle.

## Conflicts of Interest

The authors declare no conflicts of interest.

## Supporting information


**Figure S1** Pre‐penetration time of frozen section.

**Figure S2** **Subpopulations identified in myonuclei.** (A) Dimensionality reduction via t‐SNE of myonuclei in TB and Duroc pigs. t‐SNE maps showing the expression levels of myofiber type markers (*MYH7*, *MYH2*, *MYH4*, and *MYH1*) in myonuclei subpopulations. (B) Differential gene expression analysis showing the top 10 DEGs for myonuclei subpopulations. (C) Violin plot of myofiber marker genes (*MYH7*, *MYH2*, *MYH4*, *MYH1*, and *ABLIM2*) expression density in each subpopulation. (D) Pseudotime trajectories developed analysis for myogenic cell subpopulations. (E) Heatmap illustrates the DEGs dynamics of myogenic cell differentiation, the DEGs are clustered into 4 gene sets according to k‐means. (F) Difference in metabolism‐related pathway between slow (type 1) and fast (type 2a/x, 2b, and 2x) myonuclei based on pathway enrichment analysis, *Q* value < 0.05 means significant.

**Figure S3** **Characters of FAPs subpopulations.** (A) Dimensionality reduction via t‐SNE of adipogenic cells in TB and Duroc pigs, differential gene expression analysis showing the top 10 DEGs for adipogenic subpopulations. (B) Proportion of adipogenic subpopulations in LD muscle of TB and Duroc pigs, significantly different between TB and Duroc pigs (*p* < 0.1) analysed by *t*‐test. (C) Pseudotime trajectories developed analysis for adipogenic cell subpopulations. (E) Pathway enrichment analysis of DEGs for adipocytes. (D) and (F) t‐SNE maps showing the expression levels of genes related to adipogenesis and lipolysis in adipogenic subpopulations.

**Figure S4** **CellphoneDB analysis reveals complex signal communication between myogenic, adipogenic, and immune cells.** (A) and (B) Heatmap depicting the number of all possible interactions between the subpopulations of myogenic, adipogenic, and immune cells in TB and Duroc pigs, the scale bar represents the number of ligand‐receptor pairs between cell populations. (C) An interaction is indicated as colour‐filled circle at the cross of interacting cell population (x‐axis) and a ligand‐receptor pair (y‐axis), colour represents the means of the average expression level of the interacting pair analysed by CellphoneDB.

**Figure S5** **Characterizing LD muscle tissue in TB and Duroc pigs using spatial transcriptome.** (A) Visium spots spatially plotted and coloured by cluster based on expression profile of spots in TB and Duroc pigs. (B) UMAP map of the Visium spots coloured by clusters for TB and Duroc pigs. (C) Heatmap presents top 8 DEGs for clusters of spots in TB and Duroc pigs.

**Figure S6** **Differential secretory expression profile of FAPs contributed to microenvironment in LD muscle.** (A) Volcano plot of differential gene analysis between FAPs in TB and Duroc pigs (snRNA‐seq), red represents up‐regulated DEGs, blue represents down‐regulated DEGs. (B) KEGG enrichment analysis of DEGs in TB vs Duroc (FAPs from snRNA‐seq). (C) Secretory DEGs identified in TB vs Duroc pig (FAPs from snRNA‐seq). (D) Upregulated/downregulated secretory genes identified in subpopulations of FAPs between TB and Duroc pigs. (E) Upregulated secretory DEGs identified in subpopulations of FAPs between FAPs‐high vs FAPs‐low regions in TB and Duroc pigs.

**Figure S7** **Expression level of potential secretory DEGs (Figure 6C) in major cell populations.** (A) and (B) Volcano plot of differential gene analysis between MuSCs and myeloid cells in TB and Duroc pigs (snRNA‐seq), red represents up‐regulated DEGs, blue represents down‐regulated DEGs. (C) Expression levels of secretory genes related to energy, nitrogen, and lipid metabolism in cell populations. The colour represents the relative average expression of genes, and the size represents expressed percentage of genes in each cell populations.

**Table S1** Information of nuclei in snRNA‐seq.

**Table S2** Marker genes for cell population identification.

**Table S3** DEGs of TB vs Duroc (FAPs‐high region).

**Table S4** DEGs of FAPs‐high vs FAPs‐low region (TB).

**Table S5** DEGs of FAPs‐high vs FAPs‐low region (Duroc).

**Table S6** DEGs of TB vs Duroc (FAPs) snRNA‐seq.

**Table S7** DEGs of TB vs Duroc (myonuclei) snRNA‐seq.

**Table S8** Identification of MuSCs‐derived secretory DEGs.

**Table S9** Identification of myeloid cells‐derived secretory DEGs.

## Data Availability

All necessary data to evaluate the paper's conclusions are available in the paper and Supporting Information. The raw sequence data reported in this paper will be deposited in ScienceDB that are publicly accessible at https://www.scidb.cn/.
