# Supplementary figures and images for: snRNA‐Seq and Spatial Transcriptome Reveal Cell–Cell Crosstalk Mediated Metabolic Regulation in Porcine Skeletal Muscle
Source: J Cachexia Sarcopenia Muscle. 2025 Mar 13;16(2):e13752. doi: 10.1002/jcsm.13752 (PMC11904818; doi:10.1002/jcsm.13752)

A

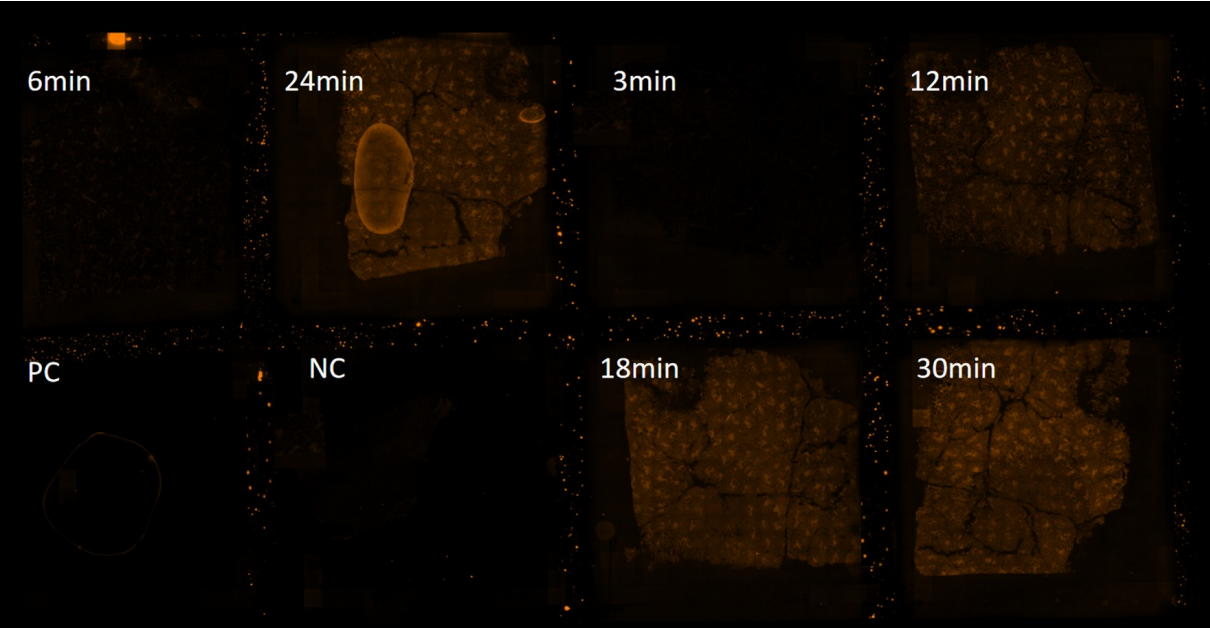

B

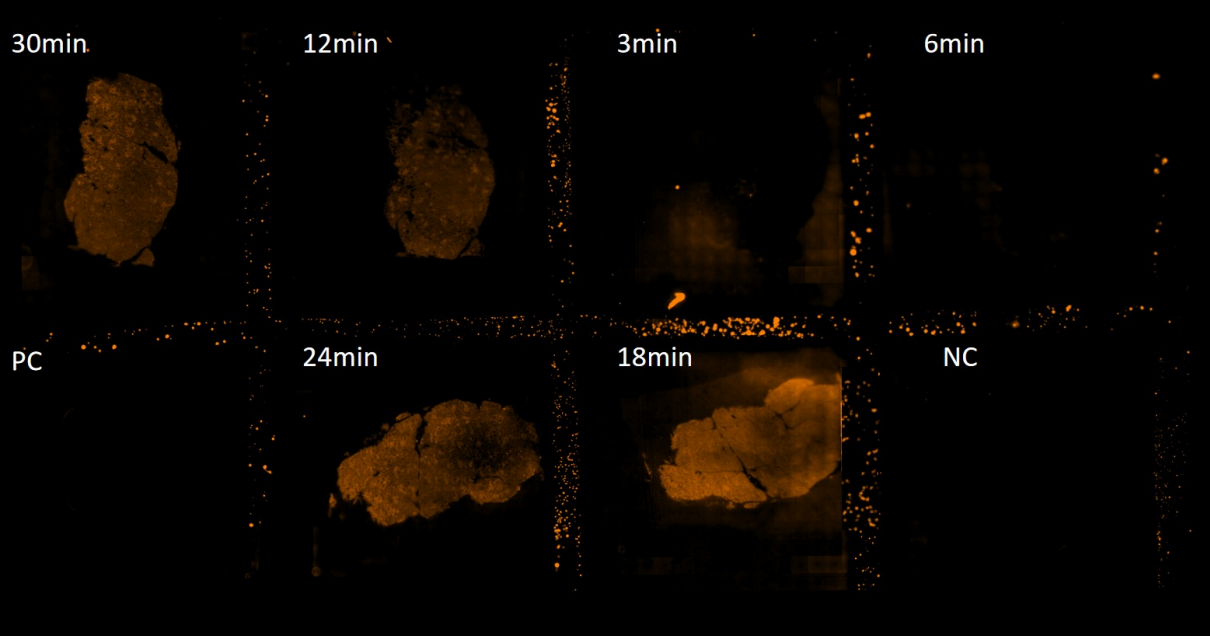

Supplement: Supplementary file 1 — Figure S1 Pre‐penetration time of frozen section. [file JCSM-16-e13752-s006.pdf]

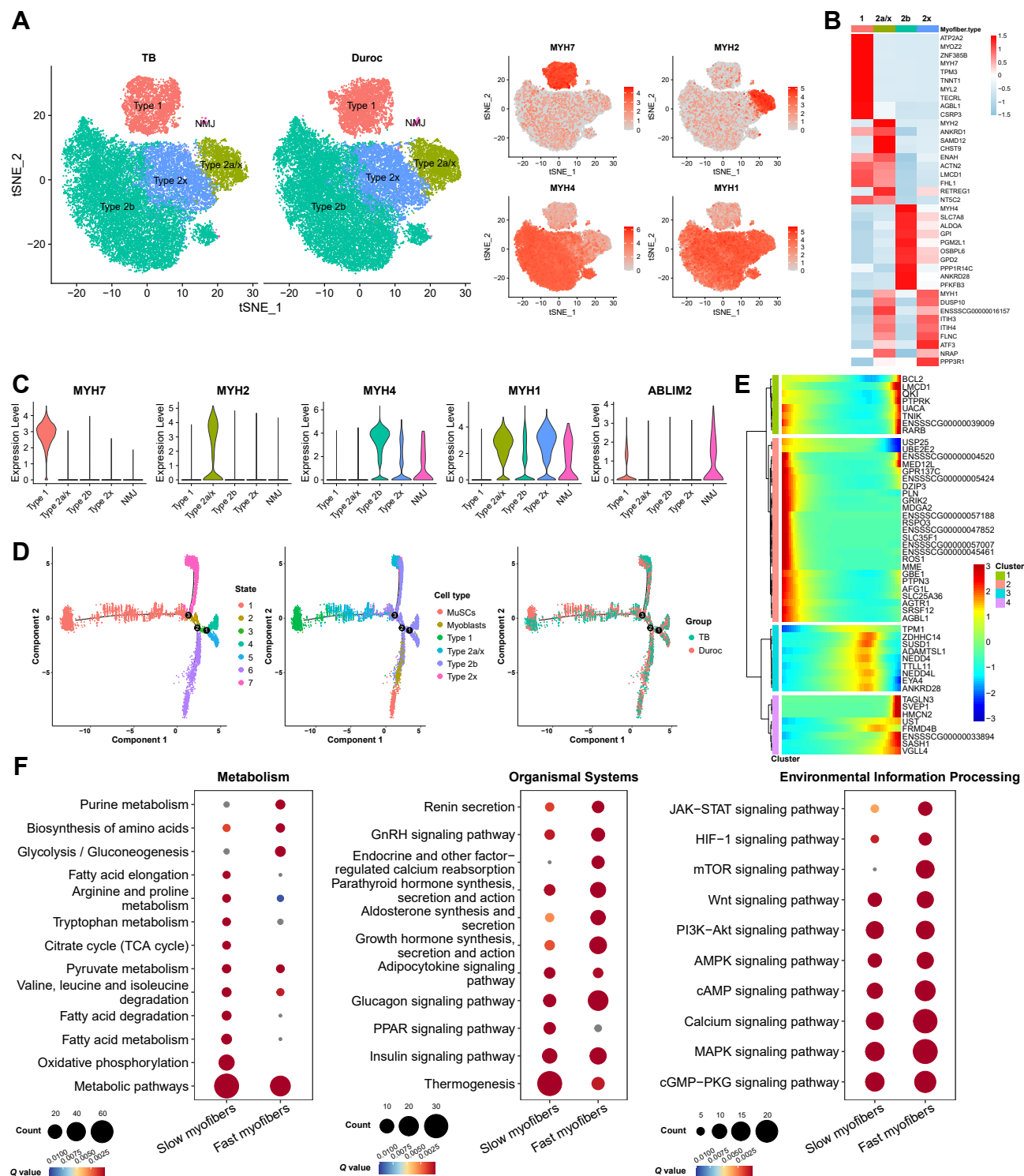

Supplement: Supplementary file 2 — Figure S2 Subpopulations identified in myonuclei. (A) Dimensionality reduction via t‐SNE of myonuclei in TB and Duroc pigs. t‐SNE maps showing the expression levels of myofiber type markers (MYH7, MYH2, MYH4, and MYH1) in myonuclei subpopulations. (B) Differential gene expression analysis showing the top 10 DEGs for myonuclei subpopulations. (C) Violin plot of myofiber marker genes (MYH7, MYH2, MYH4, MYH1, and ABLIM2) expression density in each subpopulation. (D) Pseudotime trajectories developed analysis for myogenic cell subpopulations. (E) Heatmap illustrates the DEGs dynamics of myogenic cell differentiation, the DEGs are clustered into 4 gene sets according to k‐means. (F) Difference in metabolism‐related pathway between slow (type 1) and fast (type 2a/x, 2b, and 2x) myonuclei based on pathway enrichment analysis, Q value < 0.05 means significant. [file JCSM-16-e13752-s002.pdf]

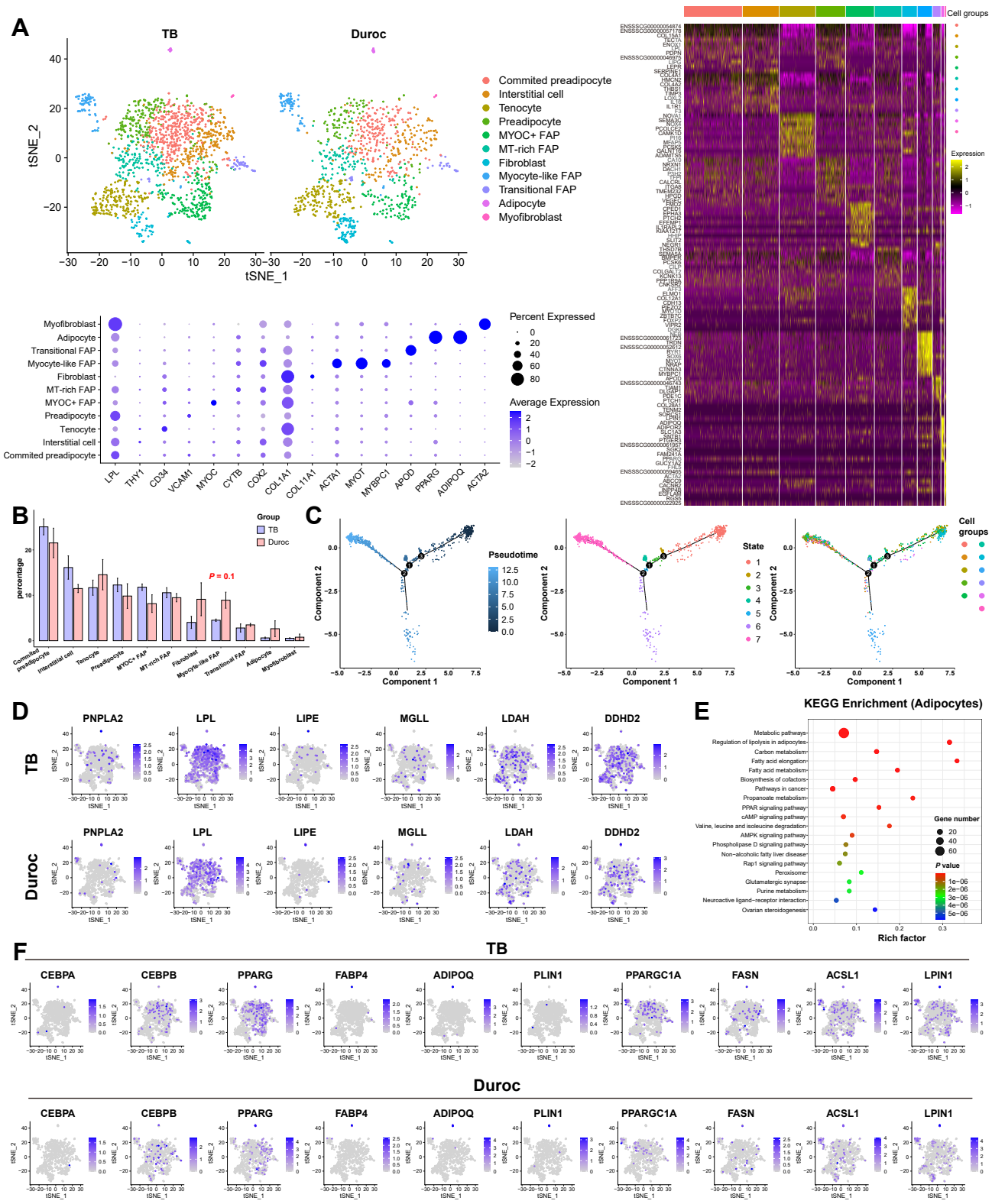

Supplement: Supplementary file 3 — Figure S3 Characters of FAPs subpopulations. (A) Dimensionality reduction via t‐SNE of adipogenic cells in TB and Duroc pigs, differential gene expression analysis showing the top 10 DEGs for adipogenic subpopulations. (B) Proportion of adipogenic subpopulations in LD muscle of TB and Duroc pigs, significantly different between TB and Duroc pigs (p < 0.1) analysed by t‐test. (C) Pseudotime trajectories developed analysis for adipogenic cell subpopulations. (E) Pathway enrichment analysis of DEGs for adipocytes. (D) and (F) t‐SNE maps showing the expression levels of genes related to adipogenesis and lipolysis in adipogenic subpopulations. [file JCSM-16-e13752-s016.pdf]

## TB pig

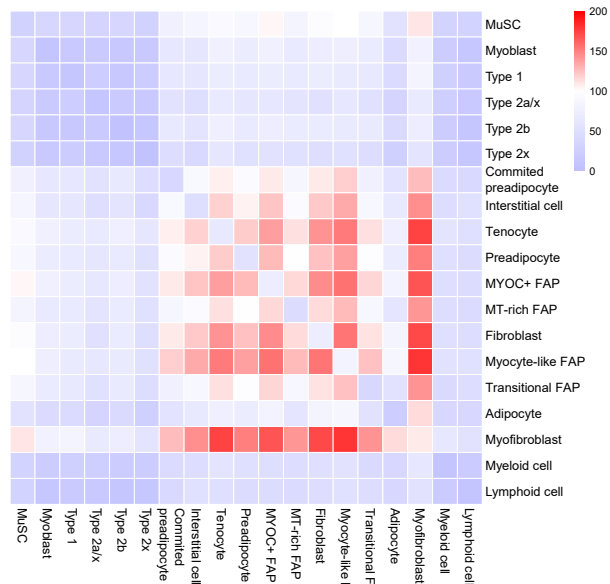

## Duroc pig

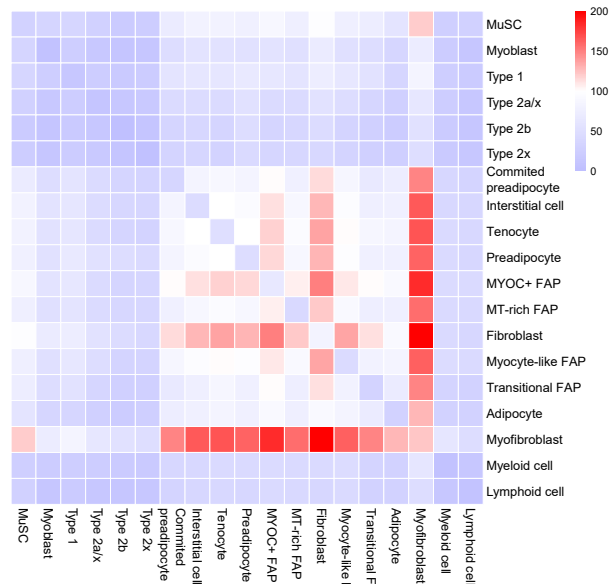

**C**

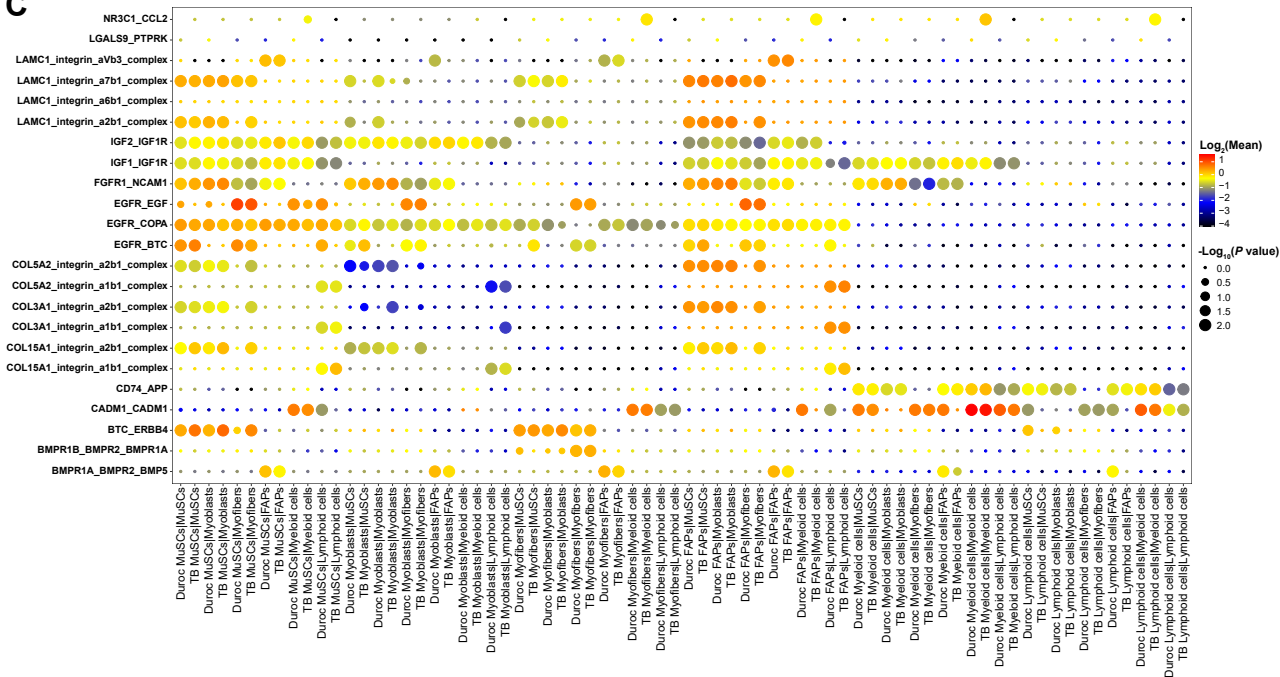

Supplement: Supplementary file 4 — Figure S4 CellphoneDB analysis reveals complex signal communication between myogenic, adipogenic, and immune cells. (A) and (B) Heatmap depicting the number of all possible interactions between the subpopulations of myogenic, adipogenic, and immune cells in TB and Duroc pigs, the scale bar represents the number of ligand‐receptor pairs between cell populations. (C) An interaction is indicated as colour‐filled circle at the cross of interacting cell population (x‐axis) and a ligand‐receptor pair (y‐axis), colour represents the means of the average expression level of the interacting pair analysed by CellphoneDB. [file JCSM-16-e13752-s015.pdf]

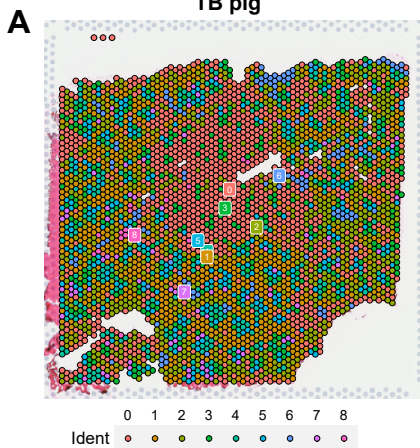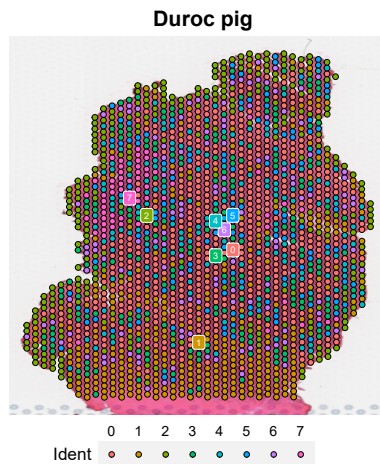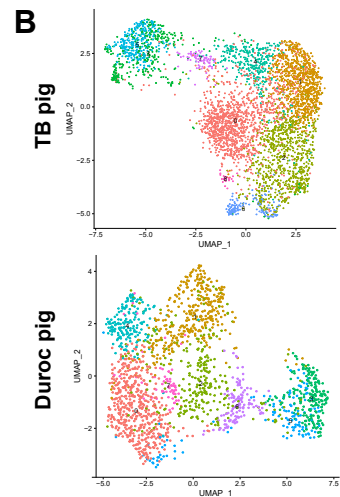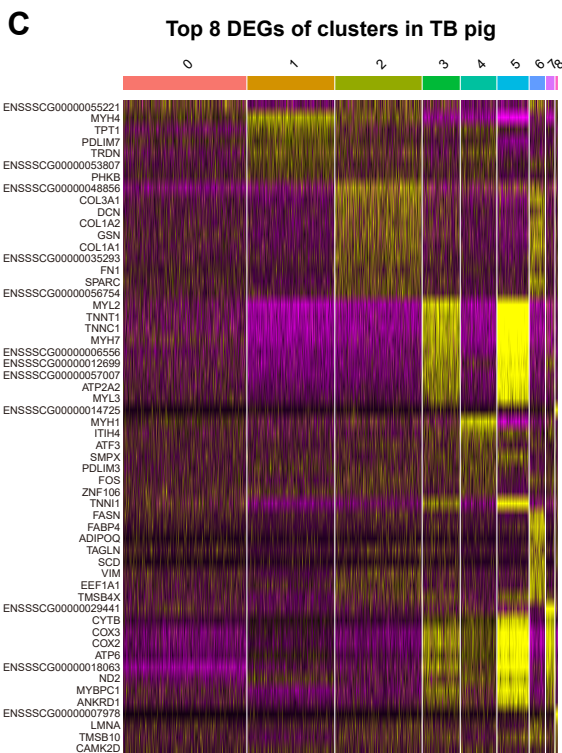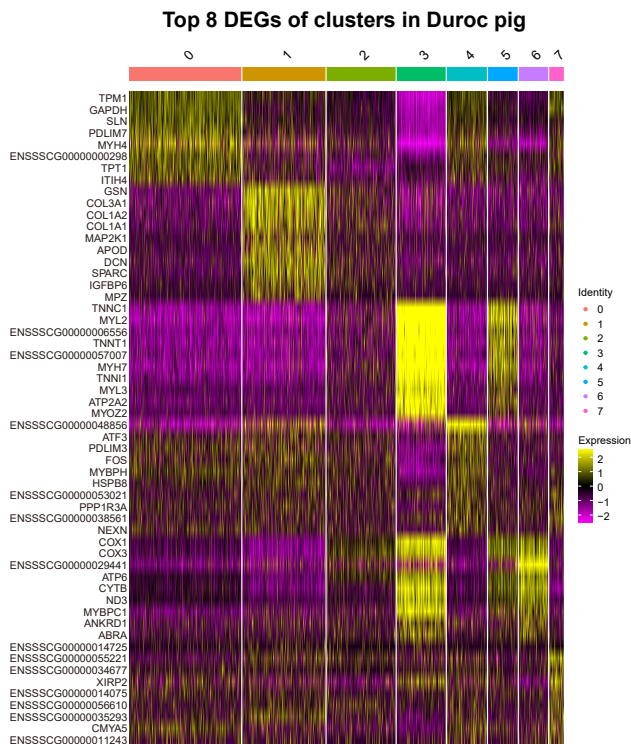

Supplement: Supplementary file 5 — Figure S5 Characterizing LD muscle tissue in TB and Duroc pigs using spatial transcriptome. (A) Visium spots spatially plotted and coloured by cluster based on expression profile of spots in TB and Duroc pigs. (B) UMAP map of the Visium spots coloured by clusters for TB and Duroc pigs. (C) Heatmap presents top 8 DEGs for clusters of spots in TB and Duroc pigs. [file JCSM-16-e13752-s012.pdf]

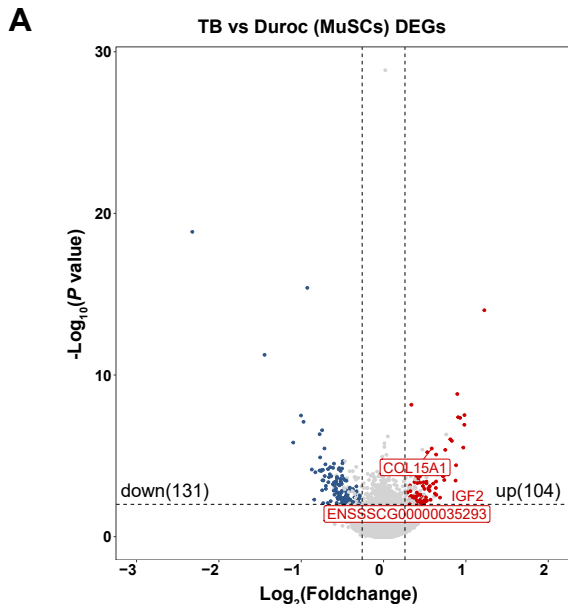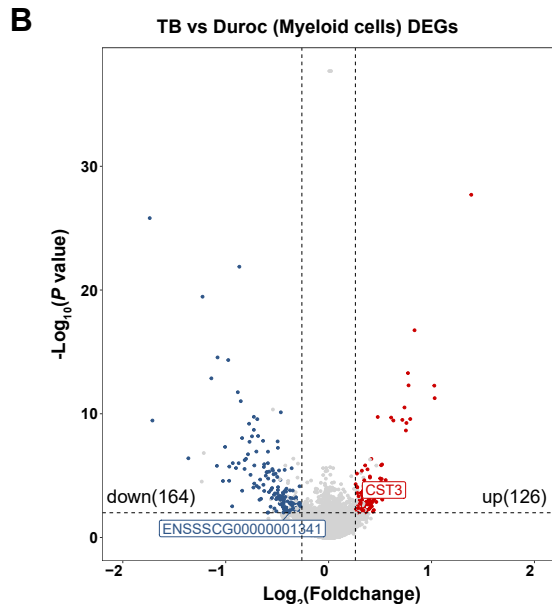

**C** Expression level of differential secreted genes in cell populations

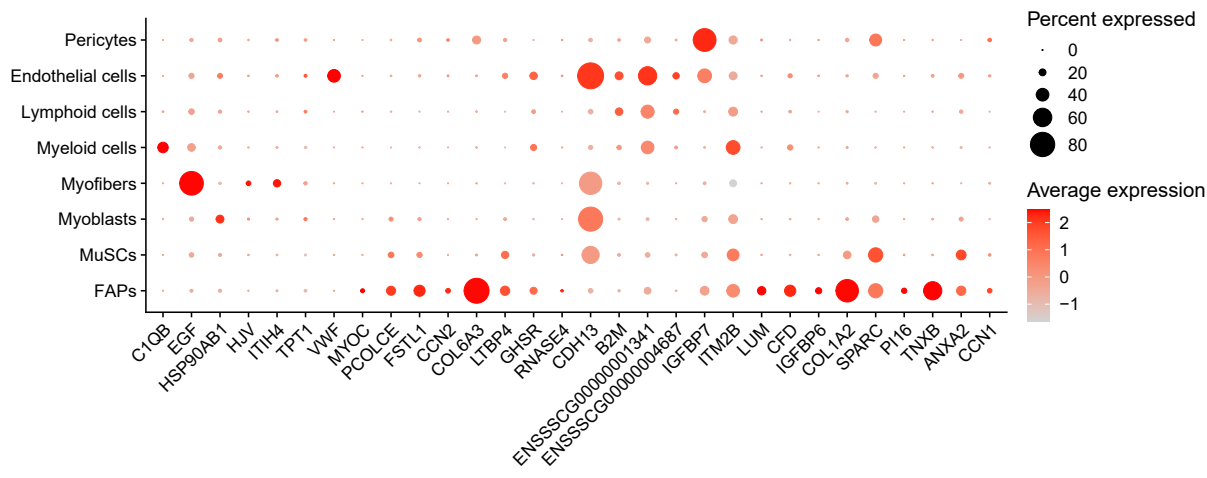

Supplement: Supplementary file 7 — Figure S7 Expression level of potential secretory DEGs (Figure 6C) in major cell populations. (A) and (B) Volcano plot of differential gene analysis between MuSCs and myeloid cells in TB and Duroc pigs (snRNA‐seq), red represents up‐regulated DEGs, blue represents down‐regulated DEGs. (C) Expression levels of secretory genes related to energy, nitrogen, and lipid metabolism in cell populations. The colour represents the relative average expression of genes, and the size represents expressed percentage of genes in each cell populations. [file JCSM-16-e13752-s007.pdf]
