# Supplementary material for: snRNA‐Seq and Spatial Transcriptome Reveal Cell–Cell Crosstalk Mediated Metabolic Regulation in Porcine Skeletal Muscle
Source: J Cachexia Sarcopenia Muscle. 2025 Mar 13;16(2):e13752. doi: 10.1002/jcsm.13752 (PMC11904818; doi:10.1002/jcsm.13752)
Supplement: Supplementary file 6 — Figure S6 Differential secretory expression profile of FAPs contributed to microenvironment in LD muscle. (A) Volcano plot of differential gene analysis between FAPs in TB and Duroc pigs (snRNA‐seq), red represents up‐regulated DEGs, blue represents down‐regulated DEGs. (B) KEGG enrichment analysis of DEGs in TB vs Duroc (FAPs from snRNA‐seq). (C) Secretory DEGs identified in TB vs Duroc pig (FAPs from snRNA‐seq). (D) Upregulated/downregulated secretory genes identified in subpopulations of FAPs between TB and Duroc pigs. (E) Upregulated secretory DEGs identified in subpopulations of FAPs between FAPs‐high vs FAPs‐low regions in TB and Duroc pigs. [file JCSM-16-e13752-s010.pdf]

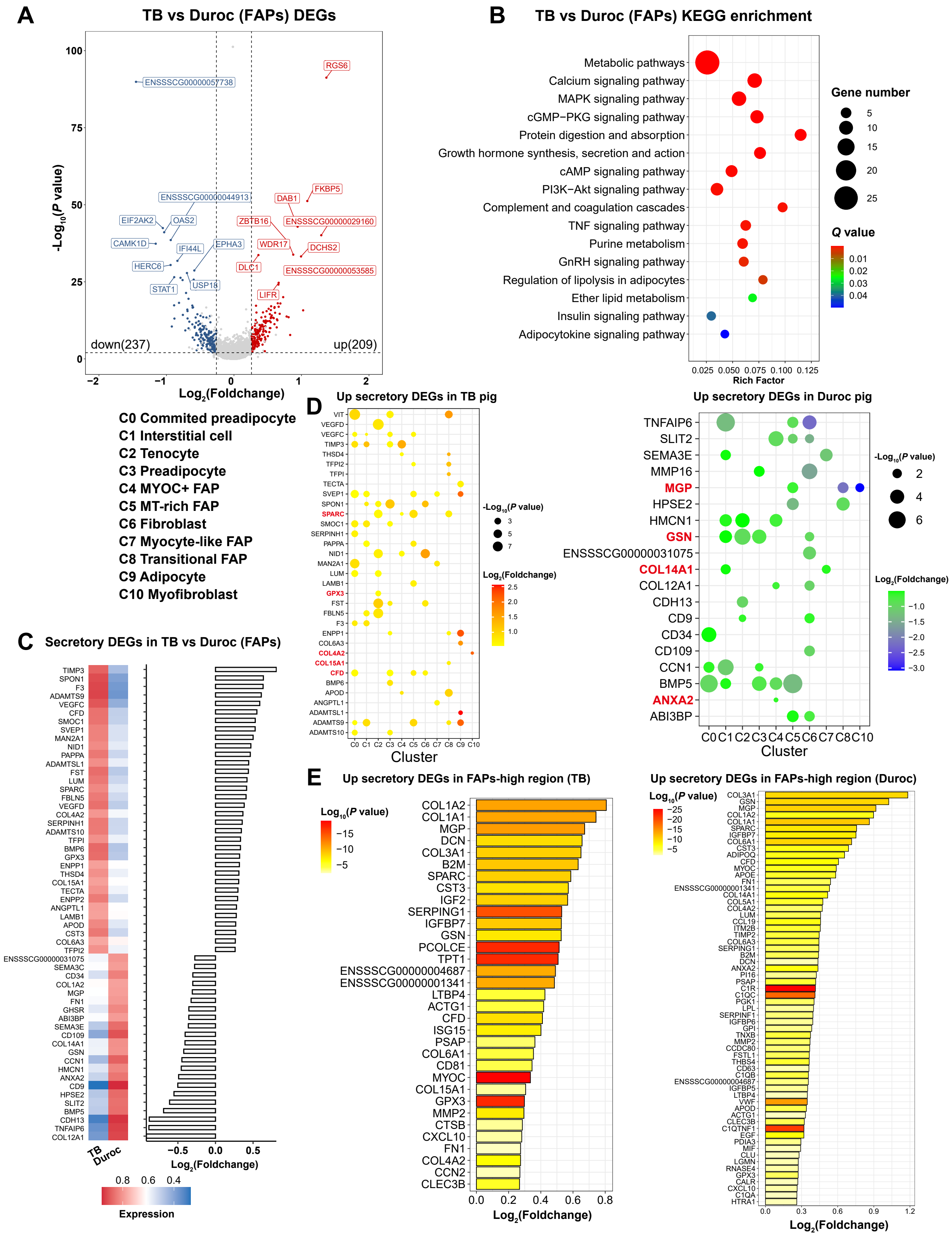
A
TB vs Duroc (FAPs) DEGs
-Log10(P value)
Log2(Foldchange)
down(237)
up(209)
RGS6
ENSSSCG00000057738
ENSSSCG00000044913
FKBP5
DAB1
EIF2AK2
OAS2
ZBTB16
ENSSSCG00000029160
CAMK1D
IFI44L
EPHA3
WDR17
DCHS2
HERC6
DLC1
ENSSSCG00000053585
STAT1
USP18
LIFR
B
TB vs Duroc (FAPs) KEGG enrichment
Metabolic pathways
Calcium signaling pathway
MAPK signaling pathway
cGMP−PKG signaling pathway
Protein digestion and absorption
Growth hormone synthesis, secretion and action
cAMP signaling pathway
PI3K−Akt signaling pathway
Complement and coagulation cascades
TNF signaling pathway
Purine metabolism
GnRH signaling pathway
Regulation of lipolysis in adipocytes
Ether lipid metabolism
Insulin signaling pathway
Adipocytokine signaling pathway
Gene number
Q value
Rich Factor
C0 Commited preadipocyte
C1 Interstitial cell
C2 Tenocyte
C3 Preadipocyte
C4 MYOC+ FAP
C5 MT-rich FAP
C6 Fibroblast
C7 Myocyte-like FAP
C8 Transitional FAP
C9 Adipocyte
C10 Myofibroblast
C
Secretory DEGs in TB vs Duroc (FAPs)
TB
Duroc
Log2(Foldchange)
Expression
D
Up secretory DEGs in TB pig
Up secretory DEGs in Duroc pig
Cluster
-Log10(P value)
Log2(Foldchange)
E
Up secretory DEGs in FAPs-high region (TB)
Up secretory DEGs in FAPs-high region (Duroc)
Log10(P value)
Log2(Foldchange)
